# Supplementary material for: Abundance-Occupancy Relationships Along Taxonomic Ranks Reveal a Consistency of Niche Differentiation in Marine Bacterioplankton With Distinct Lifestyles
Source: Front Microbiol. 2021 Jun 28;12:690712. doi: 10.3389/fmicb.2021.690712 (PMC8273345; doi:10.3389/fmicb.2021.690712)
Supplement: Supplementary file 2 [file Data_Sheet_1.pdf]

Supplementary materials for

Abundance-occupancy relationships along taxonomic ranks reveal a consistency of niche differentiation in marine bacterioplankton with distinct lifestyles

Dandan Izabel-Shen[*1,2], Anna-Lena Höger[2,3], Klaus Jürgens[2]

1. Department of Ecology, Environment and Plant Sciences, Stockholm University, Stockholm, Sweden

2 Department of Biological Oceanography, Leibniz Institute for Baltic Sea Research, Rostock, Germany

3Department of Applied Biosciences and Process Engineering, Anhalt University of Applied Sciences, Bernburger, Köthen, Germany

*Correspondence:

Dandan Izabel-Shen, dand.shen@gmail.com and dandan.shen@su.se Department of Ecology, Environment and Plant Sciences, Stockholm University, SE-106 91 Stockholm, Sweden

Contents:

Supplementary Table S1

Supplementary Table S2

Supplementary Table S3

Supplementary Table S4 (In a separate excel sheet)

Supplementary Table S5

Supplementary Table S6

Supplementary Table S7

Supplementary Figure S1

Supplementary Figure S2

Supplementary Figure S3

Supplementary Figure S4

Supplementary Table S1 Summary of the geolocation, nutrient content, bacterial cell abundances and number of sequence reads of each station. 'Sample_ID' represents the sampling station corresponding to the map (e.g., st1), size fractionation (FL, free-living or PA, particle-associated) and depth (S, surface or CIL, cold intermediate layer). Abbreviation: Temp, temperature; DD, decimal degrees; No. seqs, number of sequences; Tech_rep1 and Tech_rep 2: technical sequencing replicate 1 and replicate 2, respectively; NA: no data available. Except for the sequence reads represent size fractionated samples, abiotic and biotic variables were only differentiated for surface or CIL samples, not size fractionation related. Therefore, for example, St1_FL_CIL and St1_PA_CIL had the same salinity, and so on.

Sample_ID	Latitude DD	Longitude DD	Depth m	Tech_rep 1 No. seqs	Tech_rep 2 No. seqs	Aggregated No. Seqs	Salinity	Temp °C	Chl *a* μM/ ml	NO_3^- μM/ml	NO_2^- μM/ml	PO_4^{3-} μM/ml	NH_4^+ μM/ml	SiO_2^- μM/ml	DOC μM/ml	HNA cells/ml	LNA cells/ml
St1_FL_CIL	47.19	-59.54	65	32, 137	27, 428	59, 565	32.4	1	0.056	4.713	0.161	0.705	< 0.5	3.490	8.7	2.19E+05	1.35E+05
St1_FL_S	47.19	-59.54	3	77, 432	51, 744	129, 176	32.9	18	NA	NA	NA	NA	< 0.5	NA	NA	2.45E+06	1.57E+06
St1_PA_CIL	47.19	-59.54	65	23,476	25, 875	49, 351	32.4	1	0.056	4.713	0.161	0.705	< 0.5	3.490	8.7	2.19E+05	1.35E+05
St1_PA_S	47.19	-59.54	3	16, 954	53, 021	69, 975	32.9	18	NA	NA	NA	NA	< 0.5	NA	NA	2.45E+06	1.57E+06
St2_FL_CIL	47.83	-60.08	65	59, 113	62, 581	121, 694	32	0.44	0.147	2.796	0.121	0.716	1.247	0.691	4.9	2.07E+05	7.18E+04
St2_FL_S	47.83	-60.08	2	11, 5204	59, 537	174, 741	30.2	18.7	0.495	0.385	< 0.05	< 0.1	< 0.5	1.685	6.7	2.06E+06	1.84E+06
St2_PA_CIL	47.83	-60.08	65	30, 902	15, 028	45, 930	32	0.44	0.147	2.796	0.121	0.716	1.247	0.691	4.9	2.07E+05	7.18E+04
St2_PA_S	47.83	-60.08	2	42, 313	30, 994	73, 307	30.2	18.7	0.495	0.385	< 0.05	< 0,1	< 0.5	1.685	6.7	2.06E+06	1.84E+06
St3_FL_CIL	48.55	-62.25	50	64, 729	51, 132	115, 861	32.1	0.06	0.262	4.074	0.194	0.758	0.997	3.702	5.5	1.87E+05	1.20E+05
St3_FL_S	48.55	-62.25	6	29, 398	63, 200	92, 598	29.3	19.02	0.71	< 0.25	< 0.05	< 0.1	< 0.5	0.807	6.3	1.36E+06	1.66E+06
St3_PA_CIL	48.55	-62.25	50	25, 280	23, 728	49, 008	32.1	0.06	0.262	4.074	0.194	0.758	0.997	3.702	5.5	1.87E+05	1.20E+05
St3_PA_S	48.55	-62.25	6	22, 958	18, 905	41, 863	29.3	19.02	0.71	< 0.25	< 0.05	< 0.1	< 0.5	0.807	6.3	1.36E+06	1.66E+06
St4_FL_CIL	49.29	-63.99	50	40, 015	15, 081	55, 096	32	0.45	0.181	3.602	0.212	0.715	1.656	2.857	7.4	2.59E+05	1.77E+05
St4_FL_S	49.29	-63.99	3	56, 308	77, 259	133, 567	28.7	13.9	1.175	< 0.25	< 0.05	0.194	< 0.5	3.584	10	1.17E+06	1.66E+06
St4_PA_CIL	49.29	-63.99	50	40, 027	20, 049	60, 076	32	0.45	0.181	3.602	0.212	0.715	1.656	2.857	7.4	2.59E+05	1.77E+05
St4_PA_S	49.29	-63.99	3	17, 980	16, 009	33, 989	28.7	13.9	1.175	< 0.25	< 0.05	0.194	< 0.5	3.584	10	1.17E+06	1.66E+06
St5_FL_CIL	49.50	-66.00	50	123, 718	53, 733	177, 451	32.2	0.16	0.064	6.231	0.252	0.900	2.214	6.163	9.7	1.47E+05	1.01E+05
St5_FL_S	49.50	-66.00	3	86, 091	48, 629	134, 720	29.9	13.6	0.492	0.273	< 0.05	0.169	< 0.5	1.457	5.1	7.78E+06	9.05E+05
St5_PA_CIL	49.50	-66.00	50	38, 300	15, 989	54, 289	32.2	0.16	0.064	6.231	0.252	0.900	2.214	6.163	9.7	1.47E+05	1.01E+05
St5_PA_S	49.50	-66.00	3	2, 953	10, 386	13, 339	29.9	13.6	0.492	0.273	< 0.05	0.169	< 0.5	1.457	5.1	7.78E+06	9.05E+05
St6_FL_CIL	49.12	-67.28	55	94, 924	44, 730	139, 654	32.4	0.98	0.075	12.123	0.113	1.197	< 0.5	14.926	7.7	2.89E+05	1.50E+05
St6_FL_S	49.12	-67.28	3	30, 166	44, 612	74, 778	27.1	13.55	2.4314	1.009	0.058	0.206	< 0.5	6.858	19.6	1.76E+06	1.18E+06
St6_PA_CIL	49.12	-67.28	55	28, 440	20, 979	49, 419	32.4	0.98	0.075	12.123	0.113	1.197	< 0.5	14.926	7.7	2.89E+05	1.50E+05
St6_PA_S	49.12	-67.28	3	16, 001	8, 577	24, 578	27.1	13.55	2.4314	1.009	0.058	0.206	< 0.5	6.858	19.6	1.76E+06	1.18E+06
St7_FL_CIL	48.64	-68.63	50	58, 214	53, 552	111, 766	32.1	0.74	0.114	5.385	0.298	0.853	2.604	6.414	16.2	2.89E+05	1.50E+05
St7_FL_S	48.64	-68.63	4	43, 382	48, 980	92, 362	26.9	8.46	0.481	7.082	0.140	0.673	< 0.5	12.357	12.2	1.23E+06	5.84E+05
St7_PA_CIL	48.64	-68.63	50	30, 962	20, 055	51, 017	32.1	0.74	0.114	5.385	0.298	0.853	2.604	6.414	16.2	2.89E+05	1.50E+05
St7_PA_S	48.64	-68.63	4	19, 788	12, 779	32, 567	26.9	8.46	0.481	7.082	0.140	0.673	< 0.5	12.357	12.2	1.23E+06	5.84E+05

Sample_ID	Latitude DD	Longitude DD	Depth m	Tech_rep 1 No. seqs	Tech_rep 2 No. seqs	Aggregated No. Seqs	Salinity	Temp °C	Chl *a* µM/ ml	NO_3^- µM/ml	NO_2^- µM/ml	PO_4^{3-} µM/ml	NH_4^+ µM/ml	SiO_2^- µM/ml	DOC µM/ml	HNA cells/ml	LNA cells/ml
St8_FL_CIL	49.98	-66.23	55	41, 299	12, 562	53, 861	32.4	0.51	0.131	6.521	0.252	0.925	1.518	6.916	4.7	1.80E+05	1.34E+05
St8_FL_S	49.98	-66.23	5	71, 411	56, 127	127, 538	29.5	15.04	0.7	< 0.25	< 0.05	0.188	< 0.5	2.032	13.3	8.75E+06	6.44E+05
St8_PA_CIL	49.98	-66.23	55	32, 121	26, 153	58, 274	32.4	0.51	0.131	6.521	0.252	0.925	1.518	6.916	4.7	1.80E+05	1.34E+05
St8_PA_S	49.98	-66.23	5	24, 016	31, 562	55, 578	29.5	15.04	0.7	< 0.25	< 0.05	0.188	< 0.5	2.032	13.3	8.75E+06	6.44E+05
St10_FL_CIL	50.22	-58.47	65	47, 535	44, 129	91, 664	32.3	-0.1	0.065	8.693	0.183	1.065	0.813	10.498	5.9	1.64E+05	1.22E+05
St10_FL_S	50.22	-58.47	3	51, 873	25, 686	77, 559	30.4	16.66	0.625	< 0.25	< 0.05	0.199	< 0.5	3.633	15.5	8.31E+06	4.72E+05
St10_PA_CIL	50.22	-58.47	65	40, 809	37, 384	78, 193	32.3	-0.1	0.065	8.693	0.183	1.065	0.813	10.498	5.9	1.64E+05	1.22E+05
St10_PA_S	50.22	-58.47	3	9, 844	7, 423	17, 267	30.4	16.66	0.625	< 0.25	< 0.05	0.199	< 0.5	3.633	15.5	8.31E+06	4.72E+05

Supplementary Table S2 Wilcoxon test comparing the mean relative abundances of phyla between free-living (FL) and particle-attached (PA) lifestyles at surface and cold-intermediate layer (CIL). 'Unidentified_Bacteria' are a combination of OTUs unable to be assigned taxonomy at the phylum level. 'Others' are all OTUs assigned phyla that collectively (across samples) comprise less than 0.01 relative abundance of the total reads. This value is based on the pairwise difference between the individuals in your two groups. "V" value is based on pairwise difference between the individuals in the two groups, following a certain probability distribution. Bold values are significant at $p < 0.05$.

Free-living vs. Particle-associated lifestyles				
Phylum	Surface		CIL	
	V	p-value	V	p-value
Acidobacteria	36	0.293	34.5	0.282
Actinobacteria	75	**0.001**	51	**0.193**
BD1-5	39.5	0.48	40	0.5
Unclassified	13	**0.007**	14	**0.009**
Bacteroidetes	20	**0.039**	54	0.889
Candidate_BRC1	40	0.5	26	0.107
Candidate_division_OD1	41.5	0.56	31	0.213
Candidate_TM7	28.5	0.15	23	0.065
Chlamydiae	26	0.107	11	**0.004**
Chloroflexi	31.5	0.186	61	**0.039**
Cyanobacteria	17	**0.02**	19	**0.031**
Deferribacteres	62	**0.032**	69	**0.007**
Firmicutes	7	**0.002**	5.5	**0.001**
Fusobacteria	34	0.292	35	0.322
Gemmatimonadetes	26	0.059	22.5	0.061
Lentisphaerae	25	**0.09**	10	0.003
Planctomycetes	4	**0.014**	4	0.014
Alphaproteobacteria	67	**0.009**	74	**0.001**
Betaproteobacteria	66	**0.012**	69	**0.005**
Deltaproteobacteria	11	**0.004**	16	**0.016**
Epsilonproteobacteria	38.5	0.447	21	**0.047**
Gammaproteobacteria	52	0.17	28	0.149
TM6	34.5	0.297	26	**0.08**
Tenericutes	25.5	0.523	32	0.224
Verrucomicrobia	12	**0.005**	29	0.17
Others	38.5	0.428	22.5	0.058

Supplementary Table S3 The proportion of specialists and generalists in terms of the number of taxa at different taxonomical levels for the individual sub-communities defined by depth (surface or cold intermediate layer: CIL) and size fractionation (free-living: FL or particle-attached: PA). ‘Prop. Spec %’ and ‘Prop. Gener %’ refer to the proportion of specialists and generalists in the respective community, respectively.

Taxonomic rank	Surface			
	FL		PA	
	Prop. Spec %	Prop. Gener %	Prop. Spec %	Prop. Gener %
Species (OTU 99%)	83.96	3.06	87.74	3.63
Genus	21.28	18.96	37.11	20.87
Family	20.06	27.12	30.79	28.81
Order	18.08	30.51	29.38	35.03
Class	21.28	24.47	35.11	29.79
Phylum	17.24	31.03	20.69	44.83
	CIL			
	FL		PA	
	Prop. Spec %	Prop. Gener %	Prop. Spec %	Prop.gener %
Species (OTU 99%)	75.04	4.45	79.89	7.01
Genus	27.01	27.01	22.51	37.11
Family	21.47	37.85	18.36	47.18
Order	19.21	44.07	16.38	51.98
Class	18.09	41.49	18.09	51.06
Phylum	3.45	55.17	6.9	72.41

Supplementary Table S5 The relative abundance of specialists **A)** and generalists **B)** at the class-level for the individual sub-communities defined by depth (surface or cold intermediate layer: CIL) and size-fractionation (free-living: FL or particle-attached: PA). 'SUM' in the last row of the tables represent the sum of relative abundances of all taxa (in a column).

A) Specialist

Specialist_class	Surface_FL	Surface_PA	CIL_FL	CIL_PA
028H05-P-BN-P5(100)	0	0.000333192	0.00041649	0
Acidobacteria_unclassified(100)	0	0	0	0.000333192
Acidobacteria(100)	0	0.007413516	0.000749681	0
Actinobacteria_unclassified(100)	8.33E-05	0	0	0
AEGEAN-245(100)	0.00041649	0.000333192	0	0
A0erolineae(100)	0	0.003331917	0.00041649	0
Arctic97B-4_marine_group(100)	0.002249044	0.000749681	0	0
Ardenticatenia(100)	0	0.000333192	0	0.001166171
Armatimo0detes_unclassified(100)	0.000166596	0.006580537	0	0
Bacteroidetes_Incertae_Sedis(100)	0.000166596	0.001082873	0	0
Bacteroidia(100)	8.33E-05	0.002748832	0.00041649	0
BD2-2(100)	0	0.001332767	0	0
BD7-11(100)	0	0	8.33E-05	0.001665959
Caldilineae(100)	0	0.002082448	0	0
Candidate_division_BRC1_unclassified(100)	0.000499788	0.005331068	0	0
Candidate_division_SR1_unclassified(100)	0	0.000916277	0	0
Candidate_division_WS3_unclassified(100)	0	0	0	0.000499788
Chlorobia(100)	0	0.000916277	0	0.000249894
Chloroflexi_unclassified(100)	0	0.000333192	0	0.000749681
Clostridia(100)	0.000166596	0	0	0
Dehalococcoidia(100)	0	0	0	0.000583086
DEV055(100)	0	0.001665959	0	0.000749681
Erysipelotrichia(100)	0	0.000333192	0.000166596	0.000499788
Fibrobacteria(100)	0	0.001249469	0	0
Gemmatimo0detes(100)	0.00041649	0	0	0
Holophagae(100)	0	0.000583086	0	0
Ig0vibacteria(100)	0	0.000832979	0	0

Specialist_class	Surface_FL	Surface_PA	CIL_FL	CIL_PA
JG30-KF-CM66(100)	8.33E-05	0	0.001416065	0
JTB23(100)	0.000499788	0	0	0
Ktedonobacteria(100)	0	0.000916277	0	0
Lentisphaeria(100)	0.00241564	0	0	0
MB-A2-108(100)	0	0.001249469	0	0.000249894
Melai0bacteria(100)	0	0.001416065	0	0
ML635J-21(100)	0.001166171	0	0	0
Mollicutes(100)	8.33E-05	0	0	0
Negativicutes(100)	0	0.000333192	0	0.000666383
Oligosphaeria(100)	0	0.004997876	0	0
OPB35_soil_group(100)	0.000832979	0	0	0
OPB54(100)	0	0	0	0.000583086
PBS-III-20(100)	0	0.00041649	0	0
Pla3_lineage(100)	0.001416065	0	0	0
Planctomycetes_unclassified(100)	0.000916277	0	0.002249044	0
Proteobacteria_Incertae_Sedis(100)	0	0.000916277	0	0.00041649
R76-B128(100)	0.000166596	0	8.33E-05	0
SAR202_clade(100)	0.006330643	0.00241564	0	0
SGST604(100)	0	0	0.001166171	0
SL56_marine_group(100)	0	0.00199915	0.001332767	0.000832979
Spartobacteria(100)	0	0	0.003082023	0
SPG12-401-411-B72(100)	0	0	0.000249894	0.004248194
SS1-B-03-39(100)	0	0.005497664	0.001082873	0
Subgroup_22(100)	0	0	0	0.000583085
TM6_unclassified(100)	0	0	0.000249894	0
VC2.1_Bac22(100)	0	0.000583086	0	0
Verrucomicrobia_Incertae_Sedis(100)	0	0	0.001915852	0.000166596
Verrucomicrobia_unclassified(100)	0.001582661	0	0	0
WCHB1-41(100)	0	0.000916277	0.000249894	0
WCHB1-60_unclassified(100)	0	0.001499363	0	0
SUM	0.01974161	0.06164047	0.015326819	0.014243946

B) Generalists

Generalist_class	Surface_FL	Surface_PA	CIL_FL	CIL_PA
Acidimicrobiia(100)	5.284170894	2.084364145	2.030387085	1.511107779
Actinobacteria(100)	0.825898993	1.187162123	0.482961408	0.728273817
AEGEAN-245(100)	0	0	0.017825757	0
Alphaproteobacteria(100)	44.99979176	14.56964124	36.0935769	7.592939668
Arctic97B-4_marine_group(100)	0	0	0.128945198	0
Bacilli(100)	0	0.054310251	0.014327244	0.118699553
Bacteria_unclassified(100)	0.054976635	2.063456364	0.234733572	1.090120032
Bacteroidetes_unclassified(100)	0.439229994	3.889930113	0.368843242	2.099357773
Bacteroidia(100)	0	0	0	0.007746708
BD1-5_unclassified(100)	0	0	0.020407993	0.029654064
BD2-2(100)	0	0	0	0.00924607
Betaproteobacteria(100)	1.400071636	0.903699261	1.363920334	0.840809323
Candidate_division_BRC1_unclassified(100)	0	0	0.004914578	0.079882716
Candidate_division_OD1_unclassified(100)	0	0	0.023073527	0.03181981
Candidate_division_SR1_unclassified(100)	0	0	0	0.008912879
Candidate_division_TM7_unclassified(100)	0	0.10953678	0	0.224987713
Chlamydiae(100)	0	0.454723409	0.045314075	0.382087613
Clostridia(100)	0	0.034901833	0	0.028071403
Cyanobacteria_unclassified(100)	0.020074802	0.445810531	0.003415215	0.071636221
Cyanobacteria(100)	7.18727874	11.91443636	0.307369368	2.794895503
Cytophagia(100)	0.505118658	0.258806674	0.551598904	0.568924874
Deferribacteres(100)	0.126779452	0.022990229	0.74226787	0.030903533
Deltaproteobacteria(100)	0.607908306	2.381321272	2.617470908	5.939725616
Epsilonproteobacteria(100)	0.026322146	0.075384628	0.106288161	0.266969871
Flavobacteriia(100)	18.5661094	25.36005531	17.40401996	14.49425661
Fusobacteriia(100)	0	0.033569066	0	0.019991504
Gammaproteobacteria(100)	14.6678495	10.1932512	25.97462745	27.56228603

Generalist_ class	Surface_FL	Surface_PA	CIL_FL	CIL_PA
Gemmatimo0detes(100)	0	0	0.019075226	0.068804092
Lentisphaerae_unclassified(100)	0	0.124613706	0.005414365	0.384753147
Lentisphaeria(100)	0	0	0.064222705	0.603576813
ML635J-21(100)	0	0	0.020241397	0.018242247
Mollicutes(100)	0	0	0	0.004248195
Oligosphaeria(100)	0	0	0.01690948	0.21265962
OM190(100)	0.02457289	3.206720478	0.195583544	1.688449076
OPB35_soil_group(100)	0	0	0.021157675	0.012911179
Opitutae(100)	0.696787199	1.937509892	0.344187054	0.390417406
Phycisphaerae(100)	0.059974511	0.891787657	0.686291659	4.369976094
Pla3_lineage(100)	0	0	0.02257374	0.135442437
Planctomycetacia(100)	0.394082515	6.446593531	1.620644559	14.32249627
Planctomycetes_unclassified(100)	0	0	0	0.0443145
Proteobacteria_unclassified(100)	0.054560145	0.098957943	0.138607758	0.545934645
SAR202_clade(100)	0	0	0.300205746	0.010412241
SC3-20(100)	0.042981733	0	0.02940417	0
Spartobacteria(100)	0	0	0	0.011911604
Sphingobacteriia(100)	0.456472666	1.358755862	0.536521978	0.812071536
SPOTSOCT00m83(100)	0.095292834	0.023906506	0.201997485	0.014243946
SS1-B-03-39(100)	0	0	0	0.006913728
Thermoleophilia(100)	0	0	0	0.009079475
TM6_unclassified(100)	0	0	0	0.008829581
Verrucomicrobia_unclassified(100)	0	0	0.07854995	0.010328943
Verrucomicrobiae(100)	3.41479871	9.605417698	7.122972736	9.66289327
SUM	99.95110412	99.73161406	99.96084997	99.88221672

Supplementary Table S6 The coefficients and P-values obtained from Spearman's rank correlation analysis. P.adj indicate significant associations were subjected to Benjamini-Hochberg corrections (Benjamini and Hochberg, 1995). The multiple corrections were performed using the following code: p.adjust(p, method = "BH", n = 12) in "stats" R package, within n indicates the number of comparisons (12 comparisons for surface and CIL, separately.

	Surface					
	FL			*PA*		
	Coefficient	P-value	P.adj	Coefficient	P-value	P.adj
Phylum	0.904	9.47E-09	1.14E-07	0.936	1.11E-12	1.33E-11
Class	0.931	2.20E-16	2.64E-15	0.945	2.20E-16	2.64E-15
Order	0.893	2.20E-16	2.64E-15	0.934	2.20E-16	2.64E-15
Family	0.907	2.20E-16	2.64E-15	0.922	2.20E-16	2.64E-15
Genus	0.902	2.20E-16	2.64E-15	0.886	2.20E-16	2.64E-15
OTU	0.647	2.20E-16	2.64E-15	0.626	2.20E-16	2.64E-15

	CIL					
	FL			PA		
	Coefficient	P-value	P.adj	Coefficient	P-value	P.adj
Phylum	0.864	1.14E-07	1.37E-06	0.871	1.79E-09	2.15E-08
Class	0.931	2.20E-16	2.64E-15	0.939	2.20E-16	2.64E-15
Order	0.902	2.20E-16	2.64E-15	0.927	2.20E-16	2.64E-15
Family	0.897	2.20E-16	2.64E-15	0.935	2.20E-16	2.64E-15
Genus	0.902	2.20E-16	2.64E-15	0.938	2.20E-16	2.64E-15
OTU	0.716	2.20E-16	2.64E-15	0.719	2.20E-16	2.64E-15

Supplementary Table S7 The slope of the abundance-occupancy relationships along the taxonomic ranks **A)** and fold change of niche index between consecutive taxonomic ranks **B)** for the individual sub-communities defined by depth (surface or cold intermediate layer: CIL) and size fractionation (free-living: FL or particle-attached: PA). For each rank pair in **B)**, the value obtained from the low taxonomic rank was used as a reference value to that from the high rank.

A)

	Slope of AORs			
Ranks	Surface_FL	Surface_PA	CIL_FL	CIL_PA
Species	0.69	0.81	0.69	0.65
Genus	0.84	0.72	0.79	0.70
Family	0.81	0.73	0.80	0.72
Order	0.83	0.78	0.85	0.79
Class	0.97	0.90	0.89	0.91
Phylum	1.15	0.95	1.05	1.06

B)

	Fold change			
Rank pairs	Surface_FL	Surface_PA	CIL_FL	CIL_PA
Species -> Genus	0.217	-0.111	1.145	0.077
Genus -> Family	-0.036	0.014	0.013	0.029
Family -> Order	0.025	0.068	0.063	0.097
Order -> Class	0.169	0.154	0.047	0.152
Class -> Phylum	0.186	0.056	0.180	0.165

Supplementary Figures

Supplementary Figure S1 Non-metric multidimensional scaling (NMDS) based on Morista-Horn and generalized UniFrac matrices illustrating the beta-diversity of the particle-associated (orange) and free-living bacteria (dark olive green) in the surface water (circles) and cold intermediate layer (triangles). Filled and unfilled symbols refer to technical sequencing replicates. The Horn-Morisita distance metric (Horn, 1996) was chosen to calculate beta diversity because it is more robust to uneven samples (i.e., no normalization of the dataset) than the commonly used Bray-Curtis distance. Generalized UniFrac distance was used to compromise the biases from the unweighted and weighted UniFrac matrices, so that the distance was not dominated by highly abundant lineages (R package 'GUniFrac'; Chen et al., 2012). Closely clustered symbols indicate bacterial communities that were more similar than those further apart from each other in the multidimensional ordination.

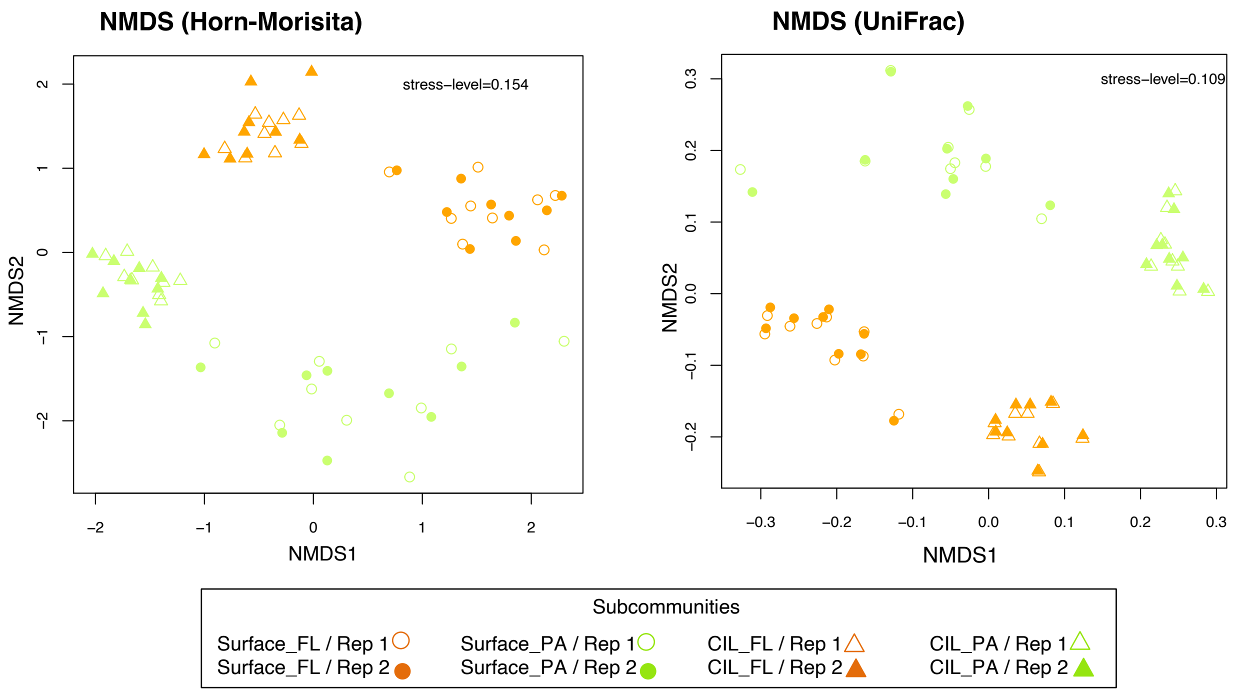

Supplementary Figure S2 Non-metric multidimensional scaling (NMDS) based on Morista-Horn illustrating the beta-diversity of the particle-associated (orange) and free-living bacteria (dark olive green) in the surface water (circles) and cold intermediate layer (triangles). Filled and unfilled symbols refer to technical sequencing replicates. The analysis was done using the exact sequence variants generated from DADA2 pipeline (Callahan et al., 2017). Raw sequences were processed using the DADA2 pipeline (Callahan et al., 2017) according to the DADA2 tutorial (v1.12) in R. The sequences were quality filtered with customized modifications as follows: truncLen=c(285,205), maxEE=4, truncQ=2, maxN=0, rm.phix=TRUE, trimLeft=c(17,22). Subsequently, denoising, merging and chimera removal were completed according to the DADA2 pipeline tutorial. All sequences were aligned and assigned taxonomically using the SILVA v.123 reference database. All archaea, eukaryote, mitochondria, and chloroplast sequences were removed. Singletons (ASVs with only one sequence read across all samples) were also discarded.

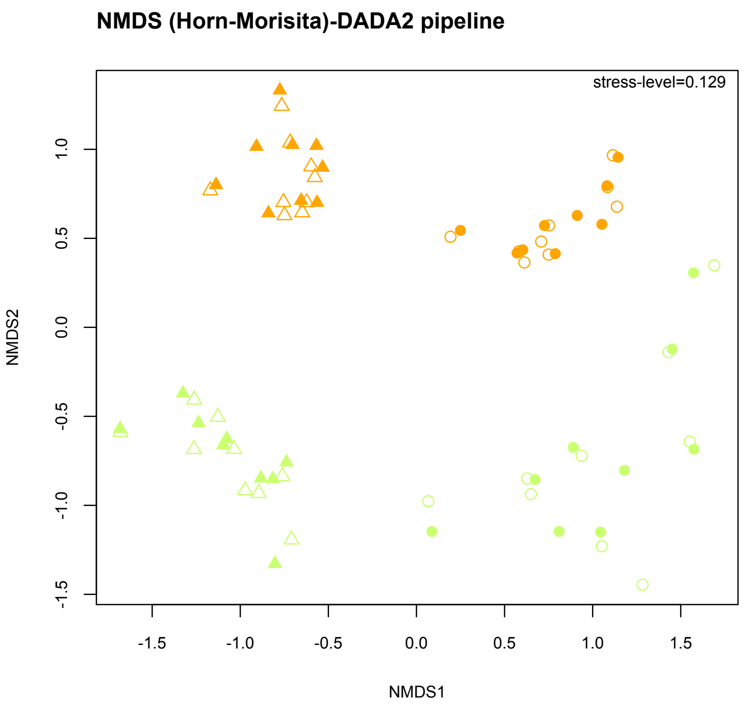

Supplementary Figure S3 Occupancy (number of sites) plotted against the mean relative abundance across taxonomic ranks from the species to thephylum level (**A–E,** except for the class level which is presented in Figure 4). The number of the occupied sites was used to define the niche breadth of bacteria. Taxa that occupied fewer than two stations were defined as habitat specialists (red), and those occupying more than five stations as habitat generalists (blue), and those not classified to either ecological group (grey). The proportion of the two ecological groups is shown on the figure. Correlation coefficient and *P* value obtained from Spearman colerration analysis are presented on the lower right corner of each panel.

A. Species level (OTU-level at 99% sequence similarity)

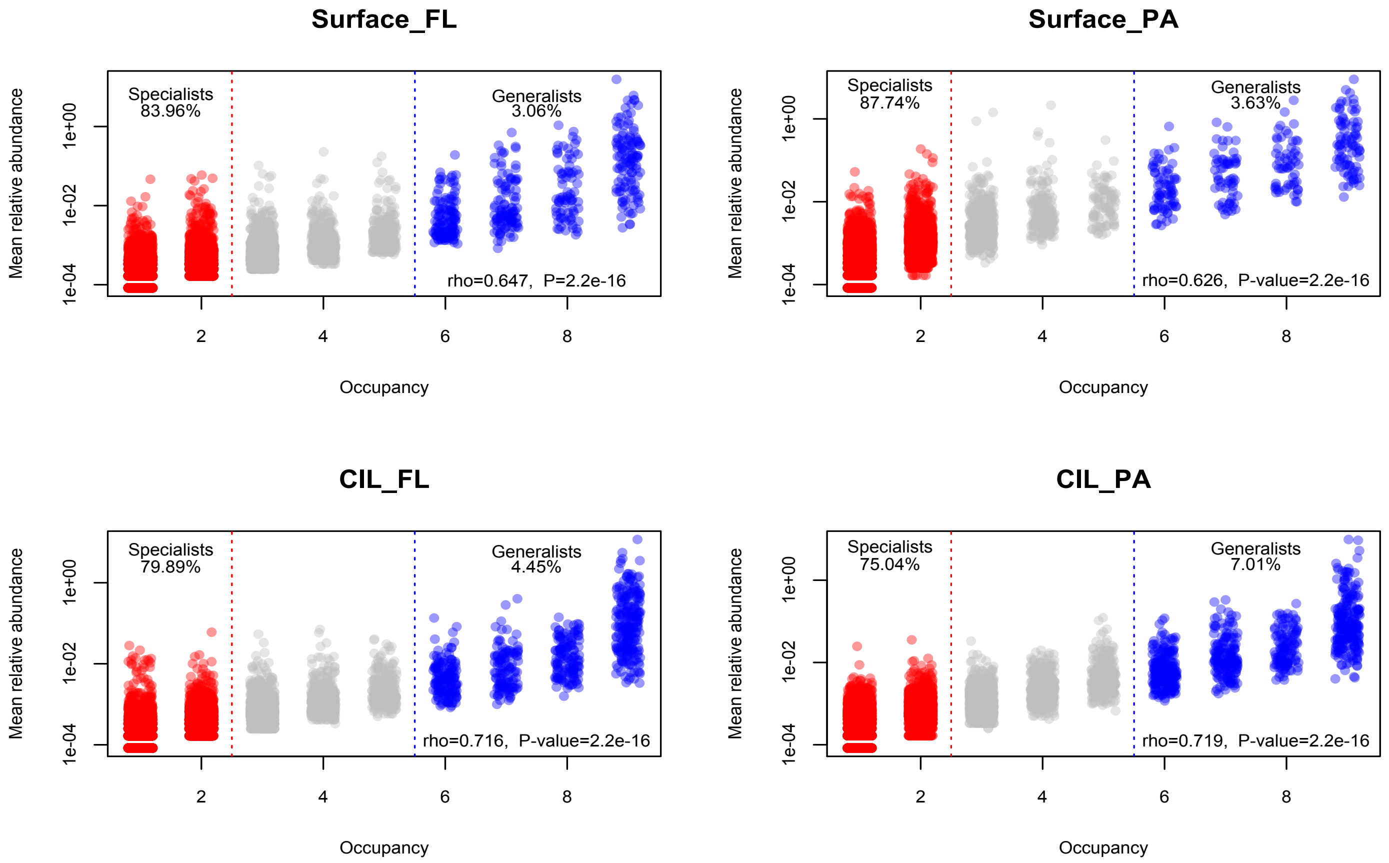

B. Genus level

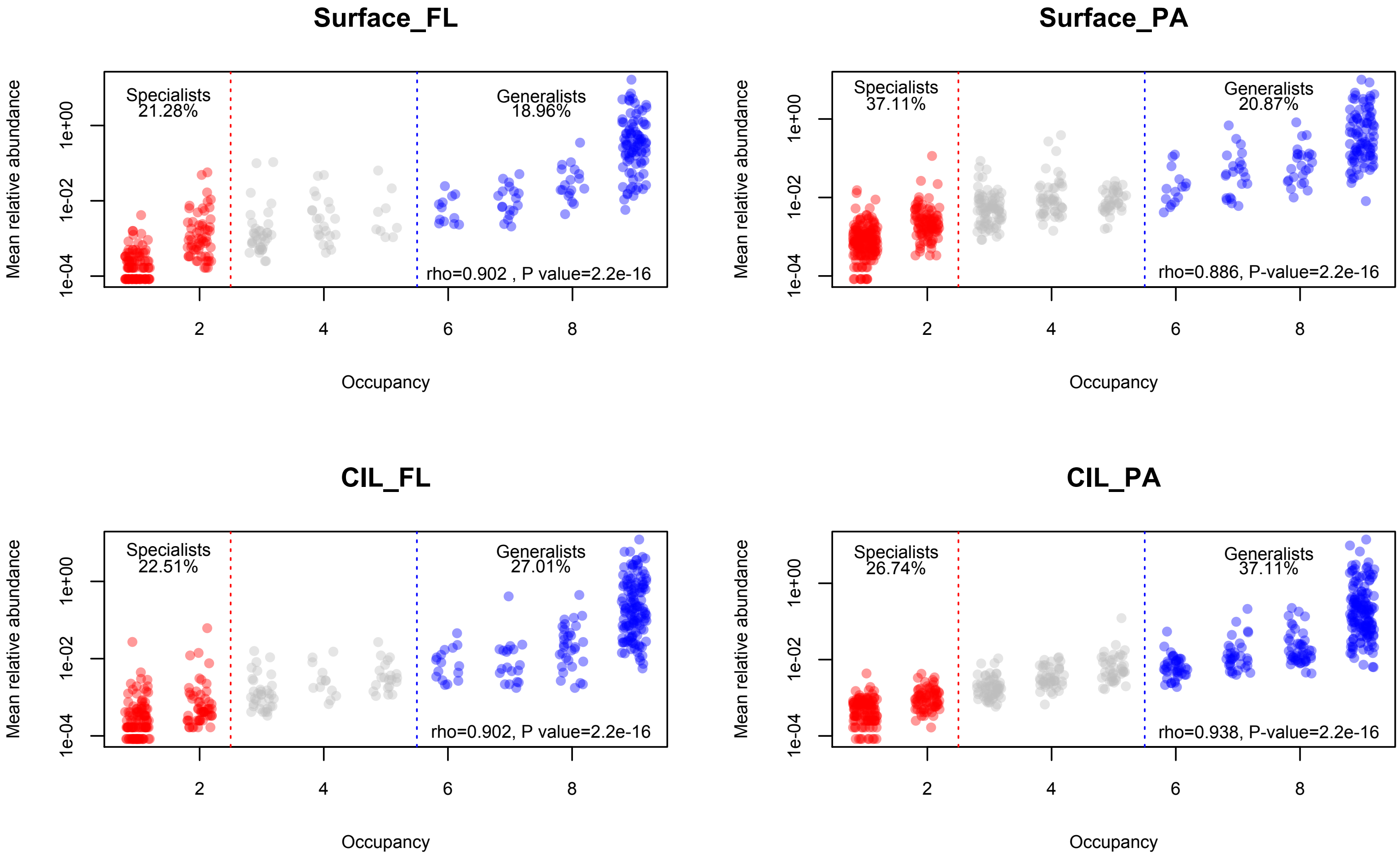

C. Family level

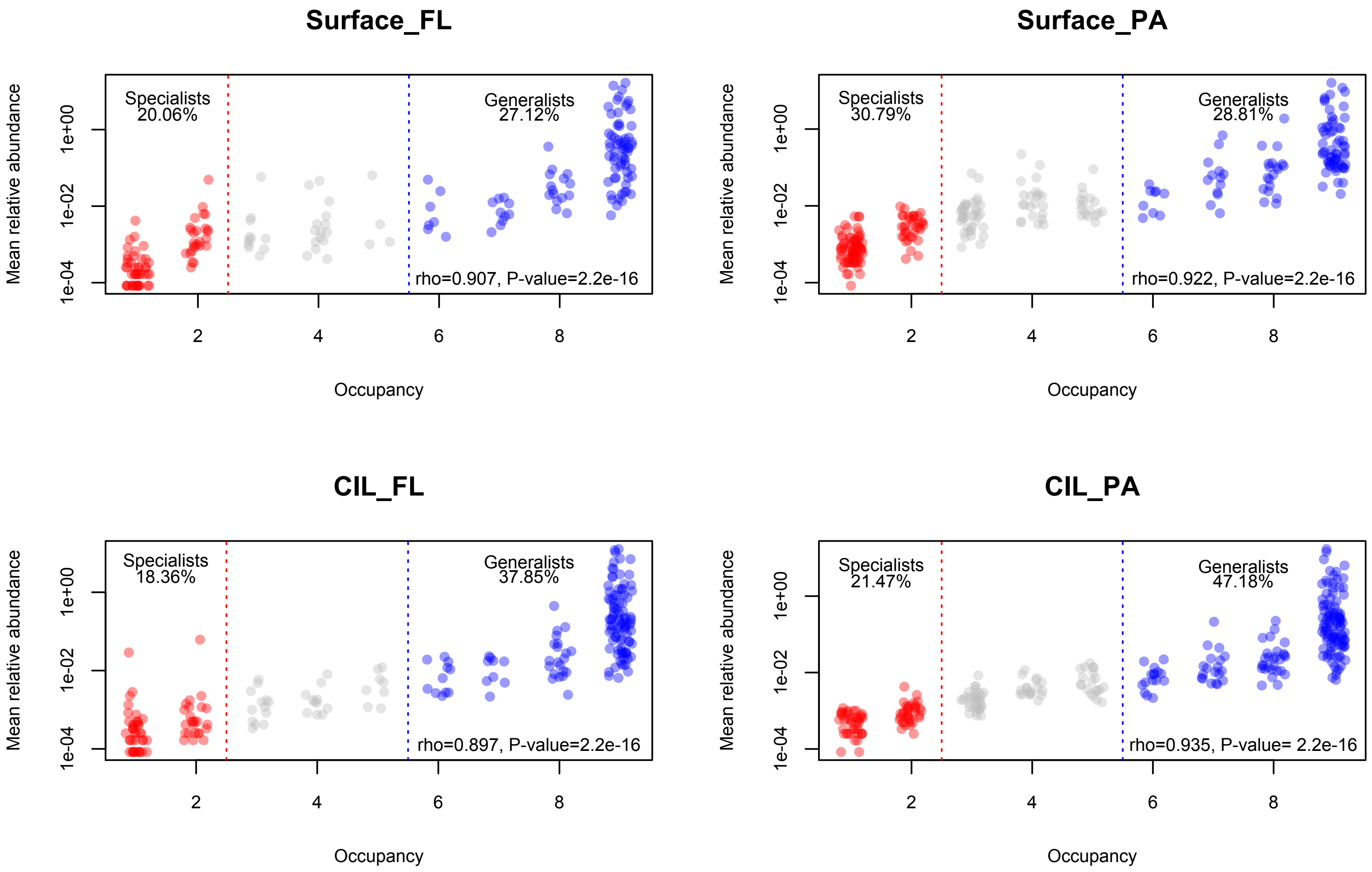

D. Order level

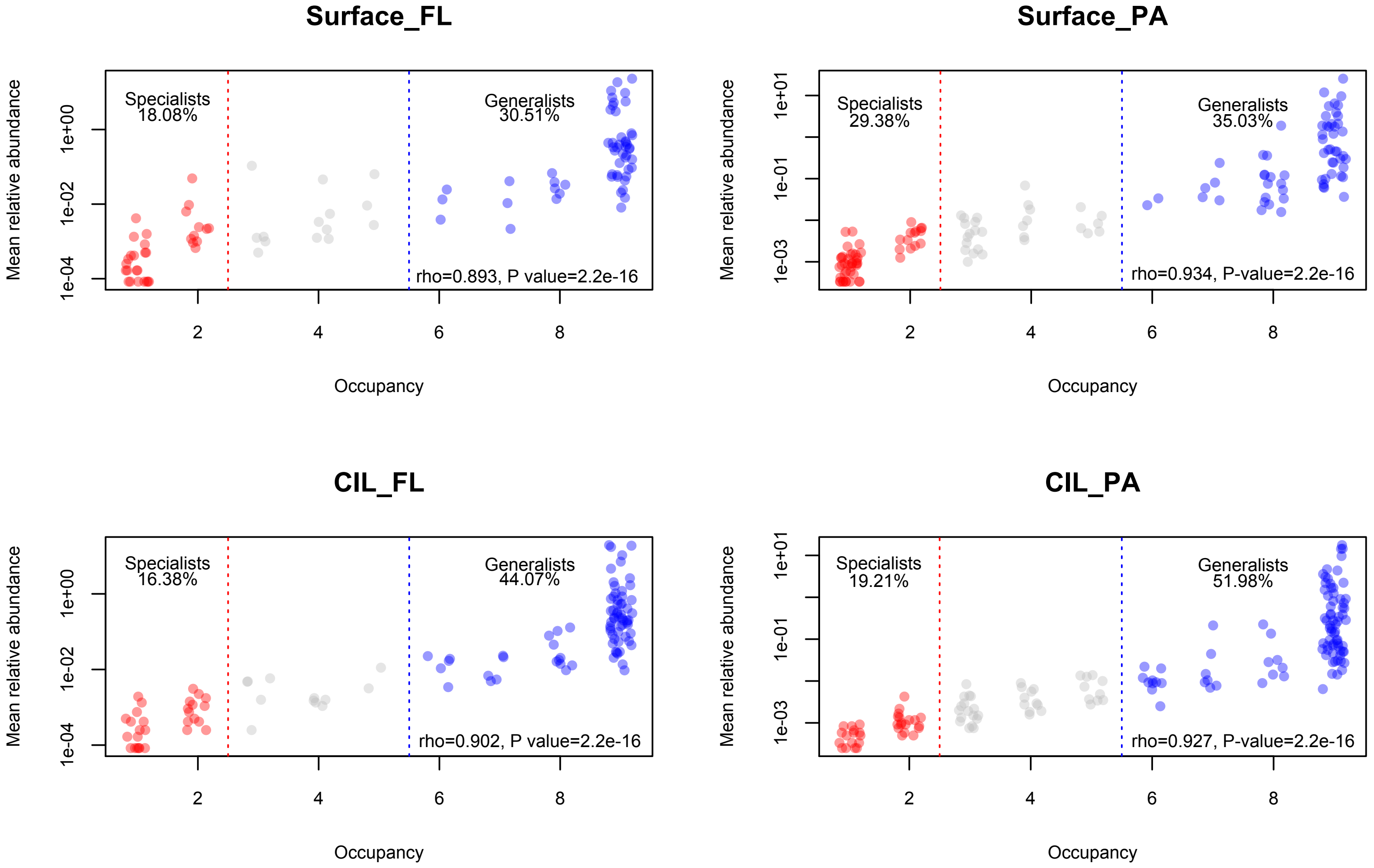

E. Phylum level

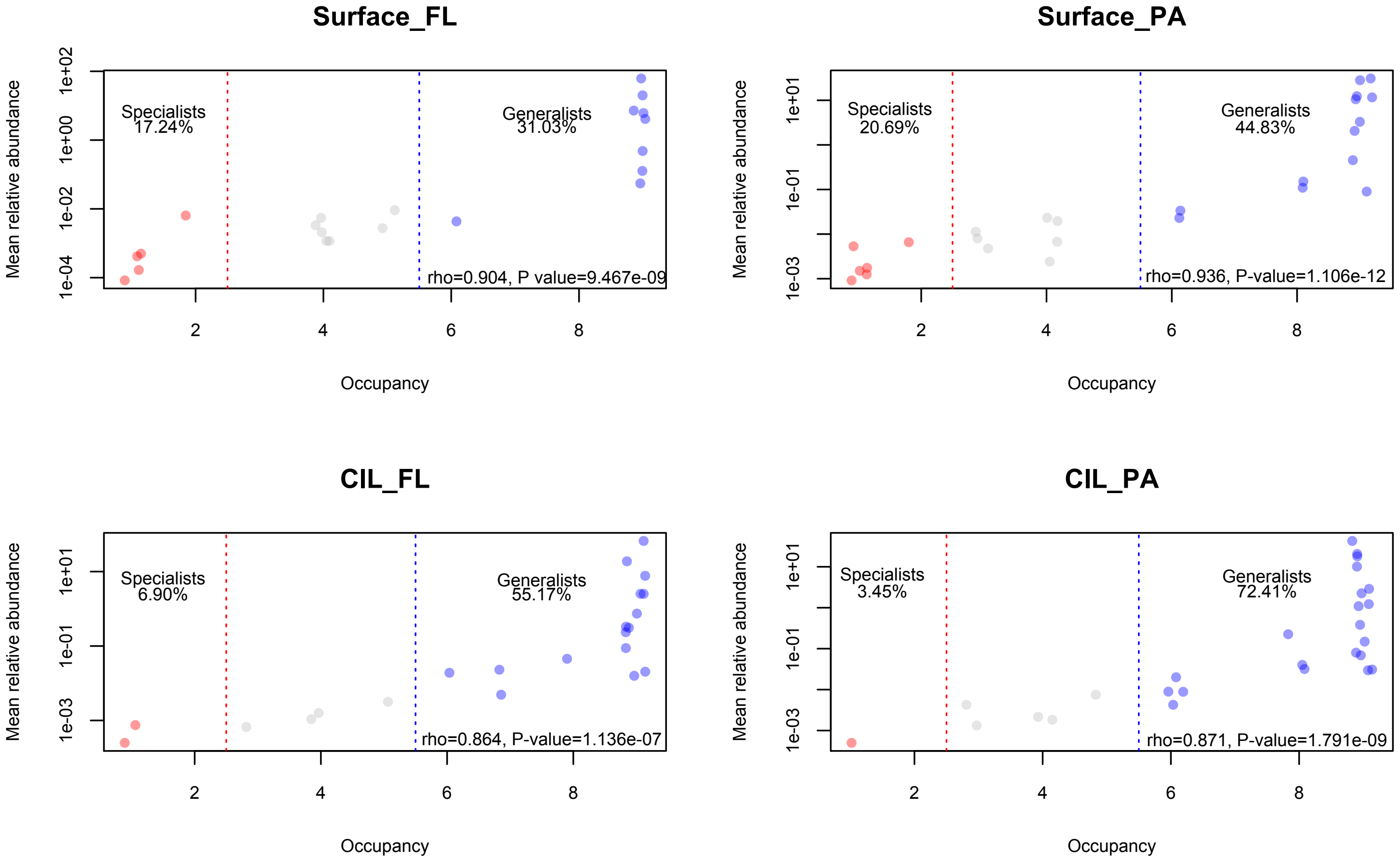

Supplementary Figure S4 Relative proportion of the top 20 bacterial classes among habitat specialists and generalists. For either ecological group, the proportion within each group was calculated from the percentage of the relative abundance of a given class / the aggregated relative abundance of the respective sample. The relative abundance of habitat specialists and generalists at the class level are given in Supplementary Table S4.

A) **B)**

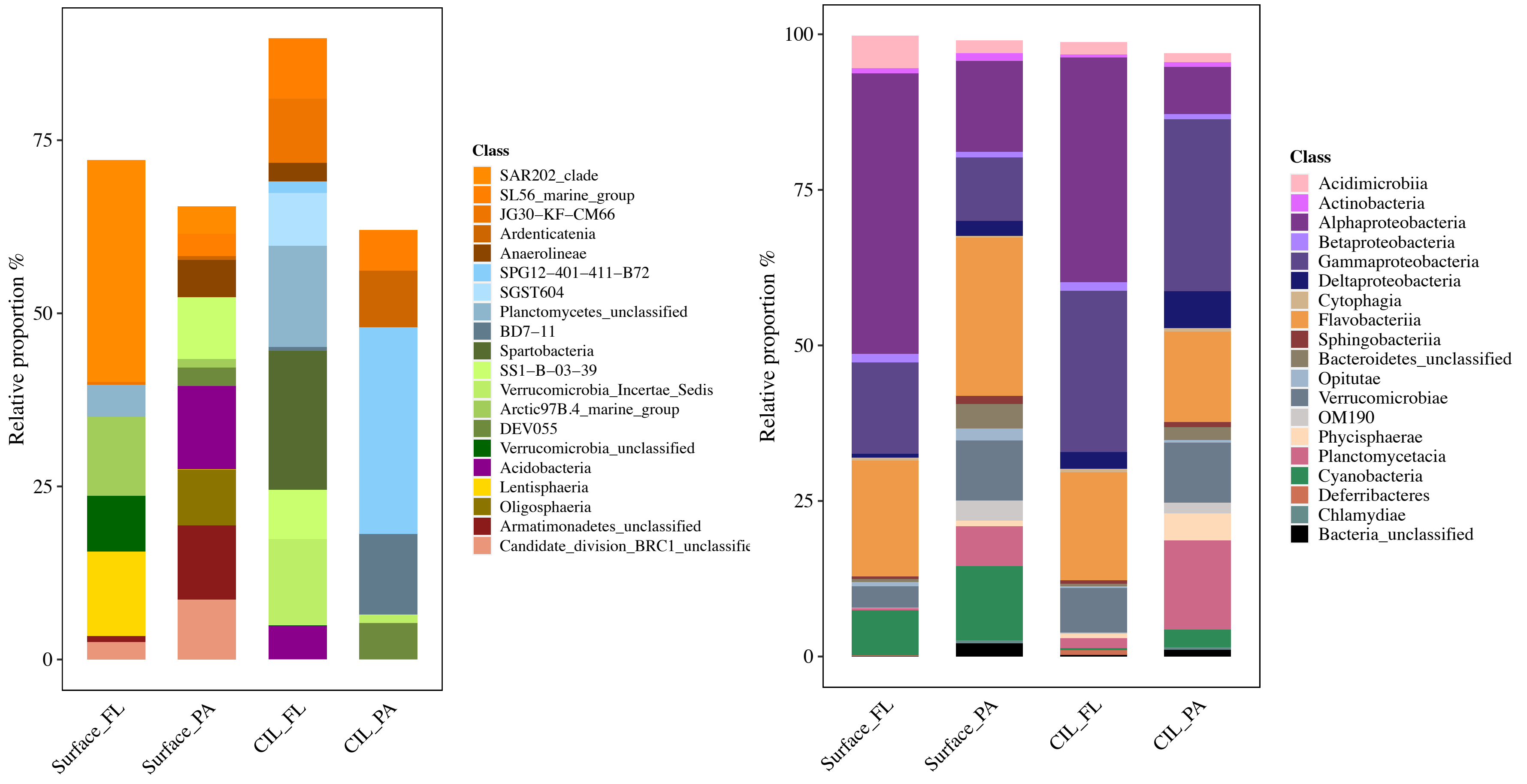

References

Benjamini Y, Hochberg Y. (1995). Controlling the false discovery rate: a practical and powerful approach to multiple testing. *J R Stat Soc* **57**: 289–300.

Callahan BJ, McMurdie PJ, Holmes SP. (2017). Exact sequence variants should replace operational taxonomic units in marker-gene data analysis. *ISME J* **11**: 2639–2643.

Chen J, Bittinger K, Charlson ES, Hoffmann C, Lewis J, Wu GD et al. (2012). Associating microbiome composition with environmental covariates using generalized UniFrac distances. *Bioinformatics* **28**: 2106–2113.

Horn H. Measurement of "overlap" in comparative ecological studies. (1996). *Am Nat* **100**: 419–424.
